# Association of *RGS4 *variants with schizotypy and cognitive endophenotypes at the population level

**DOI:** 10.1186/1744-9081-4-46

**Published:** 2008-10-03

**Authors:** Nicholas C Stefanis, Thomas A Trikalinos, Dimitrios Avramopoulos, Nikos Smyrnis, Ioannis Evdokimidis, Evangelia E Ntzani, Alex Hatzimanolis, John PA Ioannidis, Costas N Stefanis

**Affiliations:** 1 University Mental Health Research Institute, Athens, Greece; 2 Department of Psychiatry, National and Kapodistrian University of Athens, Greece; 3 King's College London, Institute of Psychiatry, Department of Psychological Medicine, London, UK; 4 Clinical and Molecular Epidemiology Unit, Department of Hygiene and Epidemiology, University of Ioannina School of Medicine, Ioannina, Greece; 5 Institute for Clinical Research and Health Policy Studies, Department of Medicine, Tufts University School of Medicine, Boston, Mass., USA; 6 Biomedical Research Institute, Foundation for Research and Technology-Hellas, Ioannina, Greece

## Abstract

**Background:**

While association studies on schizophrenia show conflicting results regarding the importance of the regulator of the G-protein signaling 4 (*RGS4*) gene, recent work suggests that *RGS4 *may impact on the structural and functional integrity of the prefrontal cortex. We aimed to study associations of common *RGS4 *variants with prefrontal dependent cognitive performance and schizotypy endophenotypes at the population level.

**Methods:**

Four *RGS4 *single nucleotide polymorphisms (SNP1 [rs10917670], SNP4 [rs951436], SNP7 [rs951439], and SNP18 [rs2661319]) and their haplotypes were selected. Their associations with self-rated schizotypy (SPQ), vigilance, verbal, spatial working memory and antisaccade eye performance were tested with regressions in a representative population of 2,243 young male military conscripts.

**Results:**

SNP4 was associated with negative schizotypy (higher SPQ negative factor for common T allele, p = 0.009; p = 0.031 for differences across genotypes) and a similar trend was seen also for common A allele of SNP18 (p = 0.039 for allele-load model; but p = 0.12 for genotype differences). Haplotype analyses showed a similar pattern with a dose-response for the most common haplotype (GGGG) on the negative schizotypy score with or without adjustment for age, IQ and their interaction (p = 0.011 and p = 0.024, respectively). There was no clear evidence for any association of the *RGS4 *variants with cognitive endophenotypes, except for an isolated effect of SNP18 on antisaccade error rate (p = 0.028 for allele-load model).

**Conclusion:**

Common *RGS4 *variants were associated with negative schizotypal personality traits amongst a large cohort of young healthy individuals. In accordance with recent findings, this may suggest that RGS4 variants impact on the functional integrity of the prefrontal cortex, thus increasing susceptibility for psychotic spectrum disorders.

## Background

Over the past few years conflicting evidence has been obtained concerning the association between the gene encoding the regulator of G-protein signaling 4 (*RGS4*) and schizophrenia. Initially evidence for linkage with schizophrenia was reported near *RGS4 *at 1q21-22 [1,2] and several association studies also suggested modest associations for certain *RGS4 *gene variants [3-9]. However, a follow-up investigation of the original linkage study found no associations with RGS4 in this same sample [10] and many other studies found also negative results. Three recent meta-analyses either show no association between *RGS4 *and schizophrenia or suggest modest effects for SNP4 (rs951436) and for two common haplotypes [11-13]. Updated evaluation of the relevant association data shows no significant association for any of the 4 most commonly studied polymorphisms [14].

A plausible hypothesis to explain the inconsistencies in these studies is that *RGS4 *variants may modulate *endophenotypes *associated with schizophrenia rather than risk of disease itself [15]. Endophenotypes are measurable components along the pathway between the genetic infrastructure and the presentation of a disorder. Candidate endophenotypes of schizophrenia with which the disorder presumably shares a degree of overlapping genetic liability include structural and morphometric brain alterations, neurocognitive deficits and schizotypal personality traits or symptoms. These are expressed as quantitative traits with different intensities across a broad phenotypic spectrum, ranging from patients to their unaffected relatives and extending to the (disease-free) general population. Recent evidence that RGS4 may indeed impact on such endophenotypes is provided by. Prasad et al [16] who reported that left dorsolateral prefrontal cortex (DLPFC) volumes were significantly different across first episode schizophrenia patients and controls on SNPs 4 (rs951436) and 18 (rs2661319) after correcting for multiple comparisons, indicating that these *RGS4 *polymorphisms may contribute to structural alterations in brain areas, previously associated with schizophrenia. Buckholtz et al [17] provided further evidence that SNP 4 (rs951436) impacts frontoparietal and frontotemporal response during working memory (n-Back test) and regionally specific reductions in gray and white matter structural volume in healthy individuals carrying the common risk allele. These studies indicate that structural and functional integrity of the prefrontal cortex might be associated with *RGS4 *polymorphic variation.

We have recently reported on the utility of adopting a population endophenotype approach to study the potential effect of candidate susceptibility genes for schizophrenia [18]. We hypothesize that endophenotypes for schizophrenia such as schizotypal personality traits [19], cognitive ability dependent on the integrity of prefrontal cortex such as sustained attention [20], working memory [21] and antisaccade eye-movements [22], may serve as potential targets of schizophrenia susceptibility genes. In this study, we set out to evaluate in a large cohort of apparently healthy young males whether 4 single nucleotide polymorphic variability in the *RGS4 *gene locus that has been previously associated with schizophrenia susceptibility, does modulate the expression of such schizophrenia related endophenotypes. We specifically hypothesized based on recent neuroimaging reports that RGS4 common "risk" alleles and haplotypes would be associated with a relative impairment of prefrontally mediated psychological and/or neuropsychological function.

## Methods

### Population

Details on the study population, assessments, and outcomes have been presented previously [18]. Briefly, the Athens Study of Psychosis Proneness and Incidence of Schizophrenia (ASPIS) examined 2243 randomly selected young male conscripts aged 18 to 24 years from the Greek Air Force in their first 2 weeks of admission to the National Basic Air Force Training Center. Conscripts underwent an extensive interview of computerized neurocognitive abilities and a self-rated psychometric evaluation. After obtaining written informed consent, DNA was extracted from mouthwash samples. This study was approved by the Bioethics and Medical Deontology Committee of the University Mental Health Research Institute.

### Assessments

The assessment battery included, among other (see below), the Raven Progressive Matrices [23] and the Schizotypal Personality Questionnaire (SPQ) [18,24,25]. Each subject performed cognitive tasks and eye-movement tasks These included assessment of verbal and spatial versions of the n-back task to assess verbal and spatial working memory [26,27] and anti-saccade eye movement task [28].

In this report we chose not to analyse other available population phenotypes that are not considered to be directly related to prefrontal function, such as general measure of state psychopathology, self rated personality scales, subclinical psychotic symptoms (Community Assessment of Psychic Experiences [29], and additional eye tracking measures.

We excluded from analyses individuals whose responses did not have face validity, based on *a priori *specified criteria. These were a negative central index of performance in 2-back tests; three or more unsuccessful trials (out of five) for verbal and spatial 2-back; and at least 50 invalid out of 90 antisaccade trials. We also excluded random responders in the TCI questionnaire [30], i.e., subjects who responded incorrectly in at least one out of four validity items.

### DNA extraction, SNP selection and genotyping

Mouthwash samples for DNA extraction were chosen as described previously [31] to obtain a better procedure acceptance rate. For *RGS4 *we selected the four SNPs reported originally by Chowdari et al [3], namely SNP1 (rs10917670), SNP4 (rs951436), SNP7 (rs951439), and SNP18 (rs2661319). These SNPs have been assessed practically in all subsequent association studies on this gene [11].

All genotyping was performed blind to phenotype measures by K-Biosciences [32] using a competitive allele-specific PCR system (CASP). Genotyping was conducted on all markers and then a quality control procedure was applied using a simple statistical exclusion protocol for removal of poor quality DNA samples: the call rate as a percentage of SNP's genotyped were determined on a sample by sample basis. DNA samples that failed to call in 60% or more SNP's were excluded from the data set to improve reliability and accuracy of the data. Regarding inter and intra plate duplication. 5.8% of the genotyping data was repeated both within 384 well PCR plates and between 384 PCR plates. This repetition showed 3 non-concordant calls indicating an error rate of < 0.1%.

### Outcomes – Statistical analysis

We quantified schizotypal traits as continuous measurements with the total score from the SPQ instrument. We assessed separately the four schizotypal latent dimensions of SPQ that emerged from applying confirmatory factor analysis to this dataset (cognitive/perceptual, negative, disorganization and paranoid factor) [25]. Moreover, we measured neurocognitive performance on four tasks: sustained attention, short-term spatial and verbal working memory and antisaccade eye movements. These measures were quantified using the corresponding sensitivity indexes: d'-CPT_IP, d'-S2B, d'-V2B and antisaccade error rate, respectively.

For every SNP we assessed compliance with the Hardy-Weinberg (HW) law using an exact test. We estimated the *D' *and r^2 ^coefficients for SNP pairs using Haploview 3.2 [33]. Haplotypes (SNP1-SNP7-SNP4-SNP18) for each gene were reconstructed with PHASE 2.1.1 [34,35]. Convergence of the Markov Chain Monte Carlo chains in PHASE was assessed by multiple (at least 5) runs. Haplotypes with frequency < 5% were grouped in a miscellaneous ("rare") category.

We checked for systematic differences in genotype and haplotype frequencies between those conscripts who were finally eligible and those who were excluded based on their random or incomplete questionnaire responses. Differences in the distributions were tested with chi-squared tests for individual SNPs and with a permutation test implemented in PHASE for haplotypes [34].

We assessed the impact of genetic factors on phenotype with regression models. Both single polymorphism analyses and haplotype-based analyses were performed. In single polymorphism evaluations, the distribution of each outcome per genotypic group was assessed with analysis of variance (ANOVA); this is a model-free approach that does not presume a specific model of genetic effect. We also examined with linear regressions whether the number of minor allele copies was associated with each outcome (allele-load or allele-based additive models). In haplotype-based assessments, we also relied on a likelihood ratio test (LR p value) to assess whether a regression model taking into account the genetic factors provided better fit (explained data better) than a constant only model. In the regression models, the most common haplotype was used as the reference category. This choice was arbitrary but is mathematically equivalent to choosing any haplotype as the reference category. In addition we contrasted the most common haplotype against all other haplotypes combined. Sensitivity analyses weighted each haplotype according to its probability as calculated by PHASE.

The main analyses did not adjust for non-genetic factors. However, in a secondary analysis, we adjusted for age and IQ and their interaction. In the latter case, we based inferences on the presence of genetic effects by assessing with a likelihood ratio test whether a model that takes age, IQ and gene haplotypes into account provides better fit than a model based on age and IQ only.

Association analyses were performed in Intercooled Stata 8.2 [36]. The reported p-values are 2-tailed and uncorrected for multiple comparisons.

For associations that passed the threshold of statistical significance at the p = 0.05 level, we also estimated the respective Bayes factors. We followed the method of Ioannidis [37] where the prior is specified as a spike null and smear alternative. The spike is placed at the null and the smear is normally distributed around the null. The Bayes factor can be readily estimated from the observed z-value (standardized effect) of the associaiton, the variance of the observed effect, and the assumed average value of the genetic effects in a given direction under the alternative. We applied these calculations illustratively for significant association in the allele-based model. The inverse of the Bayes factor shows how many times the odds that an association is true increases compared to the prior odds before running the study.

## Results

### Descriptive data

In total, 2243 randomly selected young male conscripts (mean age, 20.7 +- 1.90) entered the study. As previously described in more detail [18] the proportion of conscripts who gave eligible responses and measurements varied between 60% for SPQ and 90% for cognitive measurements.

Genotyping was successful for 81.4% on rs10917670, 88.2% on rs951436, 89.8% on rs951439, and 78.7% on rs2661319. Genotype availability was not significantly related to any of the instrument scores (p-values ranged between 0.64 and 1.00 for all analyses).

Genotype frequencies were as follows. For rs10917670: AA = 325, AG = 842, GG = 522; for rs951436 GG = 374, GT = 927, TT = 531; for rs951439: AA = 353, AG = 949, GG = 563; and for rs2661319: GG = 379, AG = 813, and AA = 441. No significant deviations from the HW law were seen for any of the 4 tested polymorphisms (p > 0.19 for all).

Strong linkage disequilibrium was observed between rs10917670 and rs951439 (r^2 ^= 0.98). r^2 ^values ranged between 0.51 and 0.81 for all other SNP pairs. In accordance with previous reported studies on individuals of Caucasian origin, the most common haplotypes were GGGG (44.0%), ATAA (40.8%), and GTGA (9.9%); all other haplotypes occurred in 3% or less of the population.

### Associations with individual SNPs – model-free approach

In model-free analyses (analyses that compare outcome distributions across the three genotypes for each SNP) a single association between SNP4 (rs951436) and the negative dimension of schizotypal personality reached nominal statistical significance (p = 0.031).

### Associations with individual SNPs – allele-load models

As shown in Table 1, there were three nominally statistically significant signals when analyses used an additive allele-load model. Specifically the T allele of SNP4 (rs951436) and the A allele of SNP18 were associated with an increase in negative schizotypy (p = 0.009 and p = 0.039, respectively). The latter was also associated with an increase in antisaccade error rate (p = 0.028). Effect sizes were modest for these nominally significant findings. The three nominally statistically significant associations (p = 0.009, 0.039 and 0.028) had Bayes factors of 0.15, 0.43, and 0.30, respectively, when the genetic effect under the alternative was assumed to have an average value in the negative direction of -0.01, -0.01, and -0.02 respectively (the magnitude of the effects estimates that were observed).

**Table 1:** Effect sizes (beta) per minor allele copy for *RGS4 *gene SNPs

	**rs10917670** **per A allele copy**			**rs951436** **per G allele copy**			**rs2661319** **per G allele copy**		
**Response**	***N***	***beta***	***P*_*all*-*load *_(*P*_*ANOVA*_)**	***N***	***beta***	***P*_*all*-*load *_(*P*_*ANOVA*_)**	***N***	***beta***	***P*_*all*-*load *_(*P*_*ANOVA*_)**

*IQ (RPM)*	1470	-0.39	0.235 (0.349)	1595	0.17	0.603 (0.799)	1411	0.10	0.773 (0.715)

SPQ *total score*	1043	0.49	0.363 (0.607)	1134	-0.65	0.215 (0.455)	1004	-0.64	0.246 (0.502)

SPQ *cognitive/perceptual factor*	1041	0.00	0.553 (0.839)	1132	-0.00	0.807 (0.966)	1002	-0.00	0.514 (0.795)

*SPQ negative factor*	1041	0.01	0.184 (0.291)	1132	-0.01	**0.009 **(0.031)	1002	-0.01	**0.039 **(0.117)

SPQ *disorganization factor*	1041	0.01	0.256 (0.493)	1132	-0.01	0.266 (0.538)	1002	-0.01	0.262 (0.493)

SPQ *paranoid factor*	1041	0.00	0.980 (0.876)	1132	0.00	0.908 (0.881)	1002	0.00	0.896 (0.976)

d'-S2B *(spatial working memory)*	1434	-0.06	0.153 (0.352)	1558	-0.02	0.514 (0.650)	1384	-0.02	0.682 (0.900)

d'-V2B *(verbal working memory)*	1372	-0.05	0.124 (0.306)	1494	0.02	0.556 (0.285)	1324	0.01	0.755 (0.785)

*Antisaccade error rate*	1585	0.01	0.286 (0.565)	1731	-0.01	0.069 (0.173)	1532	-0.02	**0.028 **(0.089)

SNP, single nucleotide polymorphism; ANOVA, analysis of variance; P_all-load_, *p *value for the allele-load model; P_ANOVA_, ANOVA *p *value. Strong linkage disequilibrium was observed between rs10917670 and rs951439 (r^2 ^= 0.98). Results of rs951439 are not shown but are similar to rs 10917670.

### Haplotype-based analyses

Overall, haplotype analyses yielded consistent results with SNP-based analyses (Table 2 and Table 3). In main analyses, likelihood ratio tests suggested a non-significant relationship between *RGS4 *haplotypes and the negative dimension of SPQ (p = 0.06; Table 2). The reference haplotype GGGG (that does not include the T allele of SNP4 and the A allele of SNP18) was associated with *lower *values in the negative dimension of SPQ, compared to the common ATAA and GTGA haplotypes (p = 0.03; Table 2), or compared to all other haplotypes combined (p = 0.01; Table 3), albeit with modest effects. In addition, non-significant trends for a relationship of haplotypes with spatial working memory were also observed (likelihood ratio testing, p = 0.07; Table 2). Adjusted analyses were qualitatively similar for the aforementioned outcomes.

**Table 2:** Haplotypes based on best pairs (unweighted) UNADJUSTED

		**Rare (< 5%)**		**ATAA**		**GTGA**		
**Response**	**N**	***b***	***se***	***b***	***se***	***b***	***se***	***LR P-value***

*IQ (RPM)*	1703	-0.626	(0.792)	-0.366	(0.332)	0.265	(0.527)	0.50

*SPQ total score*	1211	1.214	(1.279)	0.683	(0.539)	0.424	(0.881)	0.55

*SPQ cognitive/perceptual factor*	1211	0.003	(0.012)	0.003	(0.005)	-0.001	(0.008)	0.91

*SPQ negative factor*	1211	0.012	(0.013)	0.011*	(0.005)	0.020*	(0.009)	0.059

*SPQ disorganization factor*	1211	0.026	(0.021)	0.012	(0.009)	0.004	(0.014)	0.42

*SPQ paranoid factor*	1211	0.006	(0.019)	0.001	(0.008)	-0.007	(0.013)	0.92

*d'-S2B (spatial working memory)*	1656	-0.047	(0.091)	-0.015	(0.039)	0.146*	(0.063)	0.073

*d'-V2B (verbal working memory)*	1583	0.017	(0.077)	-0.028	(0.034)	0.015	(0.053)	0.77

*Antisaccade error rate*	1835	-0.023	(0.018)	0.013	(0.008)	0.013	(0.012)	0.10

The reference haplotype is *GGGG*

Shown are the coefficients of a regression model/copy of the corresponding haplotype. b, coefficient, expressing change in the response variable/haplotype copy; SE, standard error of the coefficient b; LR p value, p value for a likelihood ratio test against a constant-only model;

* significantly different (p < 0.05) from the reference haplotype GGGG (the most common in the population).

**Table 3:** Most Common haplotype vs all other haplotypes based on best pairs (unweighted)

	**UNADJUSTED**				**ADJUSTED for IQ age and interaction thereof**			
	**Per GGGG copy**				**Per GGGG copy**			

**Response**	**N**	***b***	***se***	***LR P-value***	**N**	***b***	***se***	***LR P-value***

*IQ (RPM)*	1703	0.262	(0.310)	0.40				

*SPQ total score*	1211	-0.677	(0.503)	0.18	1203	-0.535	(0.502)	0.29

*SPQ cognitive/perceptual factor*	1211	-0.002	(0.005)	0.63	1203	-0.001	(0.005)	0.87

*SPQ negative factor*	1211	-0.013*	(0.005)	**0.011**	1203	-0.011*	(0.005)	**0.024**

*SPQ disorganization factor*	1211	-0.012	(0.008)	0.16	1203	-0.009	(0.008)	0.26

*SPQ paranoid factor*	1211	0.000	(0.008)	0.99	1203	0.001	(0.008)	0.87

*d'-S2B (spatial working memory)*	1656	-0.012	(0.036)	0.74	1462	-0.045	(0.033)	0.18

*d'-V2B (verbal working memory)*	1583	0.016	(0.031)	0.60	1394	-0.004	(0.029)	0.88

*Antisaccade error rate*	1835	-0.010	(0.007)	0.15	1622	-0.004	(0.007)	0.60

Shown are the coefficients of a regression model/copy of the corresponding haplotype. b, coefficient, expressing change in the response variable/haplotype copy; SE, standard error of the coefficient b; LR p value, p value for a likelihood ratio test against a constant-only model.

* significantly different (p < 0.05) from the reference haplotype GGGG (the most common in the population).

Weighting for the probability of each haplotype (Table 4 and Table 5) suggested a trend for an association of haplotypes with spatial working memory (likelihood ratio tests, p = 0.048 without and p = 0.077 with adjustments). Specifically, each copy of the GTGA haplotype was associated with slightly superior spatial working memory performance (0.16 points) compared to individuals with the most common haplotype GGGG. Haplotype analyses limited to haplotypes where all SNPs were available versus including also those where some SNPs were missing, yielded similar results.

**Table 4:** Haplotypes weighting for the probability of each haplotype UNADJUSTED

		**Rare (< 5%)**		**ATAA**		**GTGA**		
**Response**	**N**	***b***	***se***	***b***	***se***	***b***	***Se***	***LR P-value***

*IQ (RPM)*	1703	-0.643	(0.794)	-0.351	(0.335)	0.314	(0.539)	0.49

*SPQ total score*	1211	1.322	(1.281)	0.657	(0.545)	0.421	(0.900)	0.54

*SPQ cognitive/perceptual factor*	1211	0.004	(0.012)	0.003	(0.005)	-0.001	(0.008)	0.92

*SPQ negative factor*	1211	0.013	(0.013)	0.011*	(0.005)	0.020*	(0.009)	0.061

*SPQ disorganization factor*	1211	0.028	(0.021)	0.011	(0.009)	0.004	(0.015)	0.40

*SPQ paranoid factor*	1211	0.007	(0.019)	0.001	(0.008)	-0.008	(0.014)	0.91

*d'-S2B (spatial working memory)*	1656	-0.049	(0.091)	-0.014	(0.039)	0.161*	(0.065)	**0.048**

*d'-V2B (verbal working memory)*	1583	0.022	(0.077)	-0.029	(0.034)	0.020	(0.055)	0.72

*Antisaccade error rate*	1835	-0.021	(0.018)	0.013	(0.008)	0.013	(0.012)	0.12

**Table 5:** Haplotypes weighting for the probability of each haplotype ADJUSTED for age, IQ and interaction

		**Rare (< 5%)**		**ATAA**		**GTGA**		
**Response**	**N**	***b***	***se***	***b***	***se***	***b***	***se***	***LR P-value***

*SPQ total score*	1203	0.741	(1.290)	0.510	(0.543)	0.517	(0.895)	0.77

*SPQ cognitive/perceptual factor*	1203	-0.001	(0.012)	0.001	(0.005)	-0.001	(0.008)	0.99

*SPQ negative factor*	1203	0.005	(0.013)	0.009	(0.005)	0.021*	(0.009)	0.071

*SPQ disorganization factor*	1203	0.018	(0.021)	0.009	(0.009)	0.005	(0.015)	0.67

*SPQ paranoid factor*	1203	0.003	(0.019)	-0.001	(0.008)	-0.007	(0.014)	0.95

*d'-S2B (spatial working memory)*	1462	0.124	(0.085)	0.018	(0.036)	0.135*	(0.059)	0.077

*d'-V2B (verbal working memory)*	1394	0.053	(0.073)	-0.004	(0.032)	0.017	(0.051)	0.87

*Antisaccade error rate*	1622	-0.025	(0.019)	0.008	(0.008)	0.002	(0.013)	0.35

The reference haplotype is *GGGG*. Shown are the coefficients of a regression model/copy of the corresponding haplotype. b, coefficient, expressing change in the response variable/haplotype copy; SE, standard error of the coefficient b; LR p value, p value for a likelihood ratio test against a constant-only model;

* significantly different (p < 0.05) from the reference haplotype GGGG (the most common in the population).

## Discussion

Our analysis has found that *RGS4 *variants exhibit some tentative signals for association with endophenotypes that might be relevant to the pathogenesis of schizophrenia. Common 'risk' alleles of SNP4 and SNP18, the SNPs that have been previously related also to reduced prefrontal cortex volume and function, were associated in the current study with an increase in negative schizotypal personality traits amongst a large population of apparently healthy young male conscripts. Haplotype analysis generally supported this association. An isolated model-specific effect of risk allele A (SNP18) on antisaccade error rate was noted. The SNPs 1 and 7 that were not associated with reduced brain volumes in a previous study [16] had no effect on cognitive or schizotypy endophenotypes.

Negative schizotypy personality traits in the general population similarly to their illness counterpart (negative symptoms in schizophrenia), are considered to be associated mostly with subtle deficits of prefrontal brain function. This is indicated by the modest but persistent correlation of this schizotypy factor to executive dysfunction and working memory deficits [38-40]. If *RGS4 *variants truly contribute to structural and functional alterations of the prefrontal cortex [16,17], the observed association of *RGS4 *risk variants with negative schizotypy may reflect such an impact on prefrontal function. Furthermore, the dose effect of risk allele A of SNP18 on negative schizotypy and simultaneously on antisaccade error rate (arguably a good index of Dorsolateral Prefrontal Cortex integrity) provides some further support to the notion that *RGS4 *risk variants are associated with subtle deficits at the information processing level surveyed by the prefrontal cortex. A similar pleiotropic effect of SNP4 on negative psychotic symptoms and tasks dependant on the integrity of prefrontal function (verbal fluency) was also noted in a recent association study [9] although these associations did not survive multiple comparison testing. On the other hand, we observed no clear effect of *RGS4 *polymorphisms on 2- Back working memory indices of performance as in a recent study [17], but nevertheless haplotype analyses offered a soft signal of association with spatial working memory.

*RGS4 *variation was associated with negative rather than positive schizotypy. These two factors may have relatively independent neurocognitive [40] neurochemical [41] and genetic [42,43] underpinnings. Negative symptoms in schizophrenia lay in an etiological continuum with their personality counterpart [44] and may have greater familiar, neurodevelopmental and possibly genetic basis than positive ones. Interestingly the first emerging association studies which include clinical and neurocognitive outcomes, also suggest associations of SNP4 or SNP18 with PANSS global psychopathology scores [9,45], negative PANSS symptoms and simultaneous association with tasks dependent of prefrontal brain function (verbal fluency) and impaired neurodevelopment (premorbid verbal IQ) [9].

If RGS4 variation has only modest effects on negative schizotypy endophenotypes, as observed in our study, this may explain the inconsistency of the previous association studies and the lack of a demonstrable strong association with schizophrenia per se, when these studies have been combined in meta-analyses [14]. Depending on the population mix and the importance that these endophenotypes may play in the disease process, some case-control studies of schizophrenia may show subtle effects with *RGS4 *variants, while others may show none. Genetic effects on schizophrenia risk may be very small on average and may require thousands of several tens of thousands of subjects to document or refute convincingly [46]. Conversely, quantitative traits such as these endophenotypes would be possible to study efficiently with relatively smaller sample sizes. Still, the importance of replicating our results in other cohorts cannot be over-emphasize [47,48]. Conclusive results are likely to require considerable sample sizes even for these endophenotypes, and in the presence of population or other heterogeneity, consistent replication may be difficult [49].

Along these lines, we acknowledge that, several, if not all, of the identified signals in our study might be false positives [50,51]. With 4 SNPs and 10 outcome variables, there are 40 sets of analyses performed, even without consideration of haplotypes. However, 2 SNPs were in almost perfect linkage disequilibrium and many of the outcomes also show high correlation with each other. Therefore, the multiplicity of comparisons is far less than implied at first sight. Moreover, our approach was to target variants of a gene that already had some indirect or direct support for involvement in the pathogenesis of schizophrenia and therefore the pre-study probability of significant associations was not negligible as in a hypothesis-free, discovery-oriented approach. Regardless, the interpretation of the modestly significant associations should be conservative. In addition, the Bayes factors obtained for the nominally significant associations are not very strong, but they should not be disregarded, given the prior evidence on potential implication of this genes and variants in related phenotypes.

We should also acknowledge that observed associations may exist due to hitherto unidentified risk variants at *RGS4 *that are in strong linkage disequilibrium with these four SNPs. All four SNPs in the associated haplotype are non-coding SNPs, but SNPs 1, 4 and 7 are located in a 5' region of the gene that may play a role in transcription regulation.

Another limitation of our study was the considerable rate of non-responders for some items, especially the Schizotypal Personality Questionnaire and the loss of some information due to failure of genotyping in some mouthwash samples. These missing data may have eroded some of the power of the study to detect statistically significant genetic effects. However, there is no reason why genotypes would be missed preferentially in participants with specific phenotypes and indeed we found no hint of such association. Similarly, we found no evidence that phenotype data were missing based on phenotype. Therefore, missing data is unlikely to have generated false-positives.

## Conclusion

Allowing for these caveats, our study suggests that SNP4 and SNP18 may affect some yet unidentified prefrontal-mediated neuronal mechanism that predisposes apparently healthy individuals to report higher scores on items related to social isolation, and interpersonal difficulties. These genetic effects seem to be modest, but they are definitely worth of further evaluation, since they would pertain to the wide general population of otherwise healthy individuals.

## Competing interests

The authors declare that they have no competing interests.

## Authors' contributions

NS participated in the study protocol, overall design and execution of the study, first drafted this manuscript. TT organized and performed the bulk of statistical analyses as well as writing up part of this manuscript. DA was involved in data collection, and organizing the genetic arm of the study as well as reviewing this manuscript. NS and IE participated in the study protocol, data collection and dataset organization prior to statistical analyses, and result interpretation of this manuscript. ET contributed to snp, gene identification, background literature organization and preparation of this manuscript. AH participated in part of the statistical analysis and preparation of this manuscript. JI overviewed statistical analysis contributing also to writing part and reviewing this manuscript. CS is responsible for the overall conceptualization and supervision of the ASPIS study. He was involved in the interpretation of the results of this manuscript. All authors have read and approved the final manuscript.
